# A streamlined method for transposon mutagenesis of *Rickettsia parkeri* yields numerous mutations that impact infection

**DOI:** 10.1371/journal.pone.0197012

**Published:** 2018-05-03

**Authors:** Rebecca L. Lamason, Natasha M. Kafai, Matthew D. Welch

**Affiliations:** Department of Molecular and Cell Biology, University of California, Berkeley, California, United States of America; University of Arkansas for Medical Sciences, UNITED STATES

## Abstract

The rickettsiae are obligate intracellular alphaproteobacteria that exhibit a complex infectious life cycle in both arthropod and mammalian hosts. As obligate intracellular bacteria, rickettsiae are highly adapted to living inside a variety of host cells, including vascular endothelial cells during mammalian infection. Although it is assumed that the rickettsiae produce numerous virulence factors that usurp or disrupt various host cell pathways, they have been challenging to genetically manipulate to identify the key bacterial factors that contribute to infection. Motivated to overcome this challenge, we sought to expand the repertoire of available rickettsial loss-of-function mutants, using an improved *mariner*-based transposon mutagenesis scheme. Here, we present the isolation of over 100 transposon mutants in the spotted fever group species *Rickettsia parkeri*. Transposon insertions disrupted genes whose products are implicated in a variety of pathways, including bacterial replication and metabolism, the type IV secretion system, factors with previously established roles in host cell interactions and pathogenesis, or are of unknown function. Given the need to identify critical virulence factors, forward genetic screens such as this will provide an excellent platform to more directly investigate rickettsial biology and pathogenesis.

## Introduction

Bacteria in the genus *Rickettsia* are obligate intracellular alphaproteobacteria that are divided into four groups—the spotted fever group (SFG), typhus group (TG), ancestral group (AG), and transitional group (TRG) [1]. They inhabit arthropods (ticks, fleas, and mites), and many can be transmitted to humans and other mammals. Pathogenic species primarily target endothelial cells in the vasculature, causing a variety of vascular diseases such as typhus and Rocky Mountain spotted fever [2]. Despite the prevalence of rickettsial diseases throughout the world, we know little about the bacterial factors required for infection and pathogenesis.

The SFG species *Rickettsia parkeri*, a tick-borne pathogen that causes a mild form of spotted fever in humans [3,4], is emerging as a model organism to study SFG rickettsial pathogenesis. *R*. *parkeri* can be studied under BL2 conditions and has animal models of pathogenesis that mimic aspects of human infection [5,6]. Furthermore, the *R*. *parkeri* life cycle closely matches that of the more virulent SFG species *Rickettsia rickettsii* [7,8], the causative agent of Rocky Mountain spotted fever. Like its more virulent relative, *R*. *parkeri* invades non-phagocytic cells and is taken into a primary phagocytic vacuole [9]. They then break out of this vacuole and enter the cytosol to replicate and grow [10]. *R*. *parkeri* and many other *Rickettsia* species also hijack the host cell actin cytoskeleton to polymerize actin tails and undergo actin-based motility [11–13]. During spread, motile *R*. *parkeri* move to a host cell-cell junction but then lose their actin tails before entering into a short (~1 μm) plasma membrane protrusion that is subsequently engulfed into the neighboring cell. The bacterium then lyses the double-membrane secondary vacuole to enter the neighboring cell cytosol to begin the process of spread again [14]. Because of its experimental tractability and the fact that its lifecycle is indistinguishable from more virulent species, *R*. *parkeri* provides an attractive system for investigating rickettsial host-pathogen interactions.

As an obligate intracellular pathogen, *R*. *parkeri* must produce virulence factors that usurp or disrupt various host cell pathways. However, due to their obligate growth requirement, it has been challenging to genetically manipulate rickettsiae to identify the key bacterial factors that contribute to infection [15]. Fortunately, recent advances have expanded the genetic toolkit that can be used in the rickettsiae, allowing us to peer more directly into the molecular mechanisms that drive rickettsial biology. Chief among these advances was the development of a *Himar1 mariner*-based transposon system for random mutagenesis of rickettsial genomes [16]. To date, smaller-scale mutagenesis studies have been completed in the TG species *R*. *prowazekii* [16–18] and the SFG species *R*. *rickettsii* [18,19].

Despite these advances, we still do not know all of the critical bacterial factors that mediate interactions with the host. Moreover, many of the genes in *R*. *parkeri* are annotated to encode hypothetical proteins, which limits our ability to rationally explore their functions. Therefore, we set out to expand the repertoire of available *R*. *parkeri* mutants using a forward genetic screen. We used the *mariner*-based transposon system [16] and developed a more streamlined protocol to rapidly isolate *R*. *parkeri* mutants that alter plaque size [14]. To date, we have isolated over 100 mutants that disrupt genes predicted to function in a variety of pathways. We have previously published our detailed analysis of three mutants–in *sca2*, *rickA*, and *sca4* [14,20]. Here, we present the full panel of mutants to demonstrate the potential and ease of developing rickettsial transposon libraries.

## Materials and methods

### Cell lines

Vero cells (monkey, kidney epithelial) were obtained from the University of California, Berkeley tissue culture facility and grown in Dulbecco’s modified Eagle’s medium (DMEM) (Invitrogen) containing 5% fetal bovine serum (FBS) at 37°C in 5% CO_2_.

### Transposon mutagenesis in *R*. *parkeri*

*R*. *parkeri* Portsmouth strain was a gift from Dr. Chris Paddock (Centers for Disease Control and Prevention). Wild-type *R*. *parkeri* were expanded and purified by centrifugation through a 30% MD-76R solution, as previously described [14]. The pMW1650 plasmid carrying the *Himar1 mariner*-based transposon [16] (a gift from Dr. David Wood, University of South Alabama) was used to generate *R*. *parkeri* strains carrying transposon insertions, as previously described [14] and reintroduced here. To isolate small plaque mutants, we implemented a small-scale electroporation protocol. A T75 cm^2^ flask of confluent Vero cells was infected with approximately 10^7^ WT *R*. *parkeri*. When Vero cells were at least 90% rounded at 3 d post infection, they were scraped from the flask. Infected cells were spun down for 5 min at 1800 x g at 4°C and resuspended in 3–6 ml of K-36 buffer. To release bacteria, infected cells were mechanically disrupted either by passing them through a 27.5 gauge syringe needle 10 times, or by vortexing at ~2900 rpm using a Vortex Genie 2 (Scientific Industries Inc.) in a 15 ml conical tube containing 2 g of 1 mm glass beads, with two 30 s pulses and 30 s incubations in ice after each pulse. This bead disruption procedure was adopted for a majority of the screen, because it was faster and reduced the possibility of a needle stick. Host cell debris was pelleted by centrifugation for 5 min at 200 x g at 4°C. The supernatant containing *R*. *parkeri* was transferred to 1.5 ml microcentrifuge tubes and spun for 2 min at 9000 x g at 4°C. Bacterial pellets were then washed three times in cold 250 mM sucrose, resuspended in 50 μl cold 250 mM sucrose, mixed with 1 μg of pMW1650 plasmid, placed in a 0.1 cm cuvette, and electroporated at 1.8 kV, 200 ohms, 25 μF, 5 ms using a Gene Pulser Xcell (Bio-Rad). Bacteria were immediately recovered in 1.2 ml brain heart infusion (BHI) medium. For infections of confluent Vero cells in 6-well plates, medium was removed from each well, and cells were washed with phosphate-buffered saline (PBS). Electroporated bacteria (100 μl) was added to each well, and plates were placed in a humidified chamber and rocked for 30 min at 37°C. An overlay of DMEM with 5% FBS and 0.5% agarose was then added to each well. Infected cells were incubated at 33°C, 5% CO_2_ for 24 h, and then to select for transformants, a second overlay was added containing rifampicin (Sigma) to a final concentration 200 ng/ml to select for transformants. Stock solutions of rifampicin were prepared in DMSO at 25 mg/ml and stored at -20°C. After at least 3 or 4 d, plaques were visible by eye in the cell monolayer, and plaques smaller or bigger relative to neighboring plaques were harvested and re-plated for further analysis, as described below.

To isolate and expand mutant strains before mapping the sites of transposon insertion, plaques were picked and resuspended in 200 μl of BHI. Medium was aspirated from confluent Vero cells in 6-well plates, and the isolated plaque resuspension was used to infect the cells at 37°C for 30 min with rocking. Then 3 ml DMEM with 2% FBS and 200 ng/ml rifampicin was added to each well, and infections were allowed to progress until monolayers were fully infected. Infected cells were isolated using mechanical disruption as described above, except that bacteria were immediately resuspended in BHI without a sucrose wash and stored at -80°C. These plaque-purified strains were then used as described below to map the transposon insertion sites.

### Semi-random nested PCR

To map the transposon insertion sites, plaque-purified *R*. *parkeri* strains were boiled for 10 min and used as templates for PCR reactions. Genomic DNA at insertion sites was amplified for sequencing using semi-random nested PCR. The first “external” PCR reaction used transposon-specific primers (ExTn1 5’-CACCAATTGCTAAATTAGCTTTAGTTCC-3’; or ExTn2 5’-GTGAGCTATGAGAAAGCGCCACGC-3’) and a universal primer (Univ1 5’-GCTAGCGGCCGCACTAGTCGANNNNNNNNNNCTTCT-3’). Univ1 has a specific sequence at the 5’ end and a random sequence near the 3’ end to allow for random annealing throughout the chromosome. The first PCR reaction yielded the “external” product that served as a template in the subsequent “internal” PCR reaction using transposon-specific primers (InTn1 5’-GCTAGCGGCCGCGGTCCTTGTACTTGTTTATAATTATCATGAG-3’; or InTn2 5’-GCTAGCGGCCGCCCTGGTATCTTTATAGTCCTGTCGG-3’) and a different universal primer (Univ2 5’-GCTAGCGGCCGCACTAGTCGA-3’). Univ2 contains the same specific sequence as Univ1, allowing for specific amplification of the external PCR product. PCR products were cleaned using ExoSAP-IT PCR Product Cleanup Reagent (Affymetrix) and sequenced using transposon-specific primers SR095 5’-CGCCACCTCTGACTTGAGCGTCG-3’ and SR096 5’-CCATATGAAAACACTCCAAAAAAC-3’. Genomic locations were determined using BLAST against the *R*. *parkeri* strain Portsmouth genome (GenBank/NCBI accession NC_017044.1).

## Results

### Design of an improved transposon mutagenesis scheme

**Fig 1:**
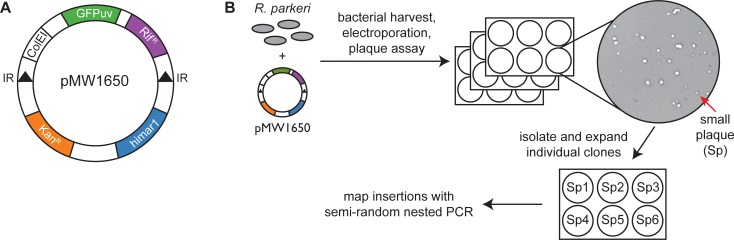
Transposon mutagenesis of *R*. *parkeri*. (A) Map of the pMW1650 plasmid used in this study for transposon mutagenesis (IR, inverted repeats). (B) Experimental scheme for transposon mutagenesis and isolation of individual mutants.

We used the pMW1650 plasmid, which carries a *Himar1 mariner*-based transposon [16], to randomly mutate the *R*. *parkeri* genome. pMW1650 encodes the *Himar1* transposase, a transposon cassette that contains the *R*. *prowazekii arr-2* rifampin resistance gene, and a variant of green fluorescent protein (GFPuv) [16] (Fig 1A). The first reported application of this system in *R*. *prowazekii* [16] and *R*. *rickettsii* [18] yielded some transposon mutants, but we sought to improve the mutagenesis scheme to increase the chances of identifying genes important for infection. Therefore, we developed a simple and rapid procedure to extract bacteria from infected host cells [14]. In the past, we had purified *R*. *parkeri* from infected host cells using an hours-long process involving mechanical disruption and density gradient centrifugation prior to electroporation [21]. In recent work, we optimized this procedure to more quickly isolate and electroporate bacteria and re-infect host cells in under an hour [14]. To mechanically disrupt infected cells, we either passed infected cells through a syringe needle or vortexed cells in the presence of 1 mm glass beads. Samples were then spun at low speed for 5 min to pellet host cell debris, followed by a 2 min high-speed spin to pellet bacteria. Bacteria were then quickly washed 2–3 times in cold sucrose prior to electroporation.

To identify genes involved in infection, we screened for transformants that showed altered plaque size and/or morphology (Fig 1B). We predicted that plaque size changes would result from defects at different stages of the rickettsial life cycle, including in intracellular growth, replication, motility, and/or spread. To screen for such mutants, pMW1650-electroporated bacteria were immediately used to setup plaque assays in the presence of rifampicin to select for transformants. Plaque size was monitored visually over the course of 3–9 days, and those displaying a small plaque (Sp) or big plaque (Bp) phenotype relative to their neighbors were selected for expansion. After independently repeating this process 7 times, 120 Sp mutant and 2 Bp mutants were selected for further analysis, as detailed below.

### Expansion and mapping of the transposon mutants

**Fig 2:**
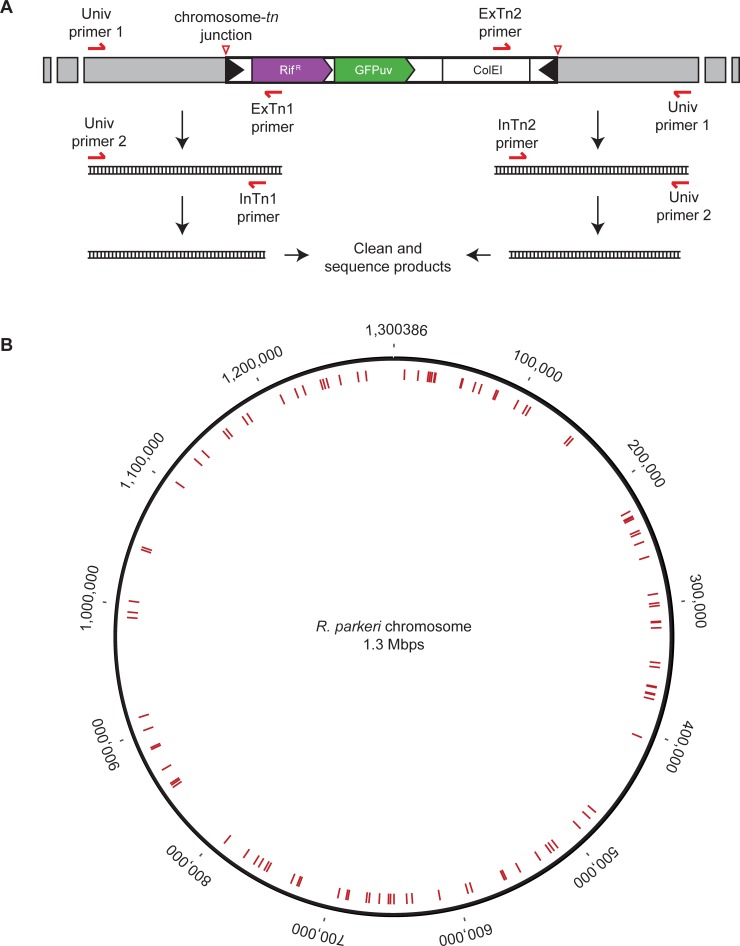
Mapping the transposon insertion sites. (A) Diagram showing the insertion of the transposon cassette into a chromosomal region (in grey). Primers specific to the transposon ends were paired with universal primers to amplify the chromosome- transposon junctions (red triangles), using semi-random nested PCR. Two nested PCR reactions were done to improve amplification of the chromosome-transposon junction directly from boiled bacteria. (B) *R*. *parkeri* chromosomal map showing all transposon insertion sites (see red lines) identified in this screen.

To expand the isolated transformants, plaques were picked and transferred to uninfected host cells to propagate each bacterial strain. Once the host cells were >75% infected, bacteria were purified using the rapid isolation procedure outlined above. Nine isolates did not grow in this expansion procedure, possibly due to a lack of live bacteria in the original plaque or poor isolation of the infected cells. The remaining transformants could be expanded, and for these the transposon insertion site was mapped using a semi-random nested PCR protocol. In short, the junctions between the transposon and the flanking genomic regions were amplified via two nested PCR reactions, using transposon-specific and universal primers (Fig 2A). PCR products were sent directly for sequencing. Mapping of the transposon insertion sites to the *R*. *parkeri* chromosome (accession number CP003341) revealed no preference for specific regions (Fig 2B), similar to what was observed in *R*. *rickettsii* [18,19] and *R*. *prowazekii* [16,18]. Using this procedure, we identified the transposon insertion sites for 106 mutants. For 6 isolates the transposon insertion site could not be mapped, and the strains did not express GFP_uv_ (data not shown), suggesting these were spontaneous rifampicin-resistant strains. Of the 106 transposon mutations mapped, 81 were within the coding regions of 75 distinct genes and 25 were in intergenic regions (Table 1). These results lay the groundwork for critical follow-up studies (e.g. purification of clonal populations, complementation, phenotypic analyses, etc.) required for revealing gene function. They also highlight that transposon mutagenesis can be readily adapted for large-scale forward genetic screening in *R*. *parkeri*.

**Table 1:** Transposon insertion sites in *R*. *parkeri*.

Name	Genome position	Gene symbol	Gene product description
Sp1	101427	MC1_00610	Putative cytoplasmic protein
Sp2	112315	MC1_00650 **	Surface cell antigen 2 (Sca2)
Sp3	681322–681323	MC1_03895	Single-stranded-DNA-specific exonuclease RecJ
Sp6	365840	MC1_02010	Cytochrome c1, heme protein
Sp7	670632	MC1_03810	Folylpolyglutamate synthase
Sp8	1047805–1047806	Intergenic	n/a
Sp9	151196–151197	MC1_00820	VirB6 Type IV secretory pathway (rvhB6e)
Sp10	491813	Intergenic	n/a
Sp11	1136147	MC1_06660	DNA polymerase I
Sp13	563189–563190	MC1_03195	RND efflux transporter
Sp14	518698–518699	MC1_02960	CTP synthetase
Sp15	520939	MC1_02980	Hypothetical protein
Sp17	1248850–1248851	MC1_07220	Transcriptional regulator
Sp18	70364	MC1_00450	Hypothetical protein
Sp19	654506–654507	MC1_03740	Antigenic heat-stable 120 kDa protein (Sca4)
Sp20	531536	MC1_03025 **	ampG protein
Sp21	20179	Intergenic	n/a
Sp22	29609	MC1_00175	F0F1 ATP synthase subunit B
Sp23	474265	MC1_02665	Outer membrane assembly protein
Sp24	753916	Intergenic	n/a
Sp25	728290	MC1_04100	Isopentenyl pyrophosphate isomerase
Sp26	33338	MC1_00210	Transcriptional regulator
Sp27	30722	Intergenic	n/a
Sp28	301811	MC1_01650	Protease
Sp29	225255	MC1_01180	Acriflavin resistance protein D
Sp30	886852	Intergenic	n/a
Sp31	262955–262956	MC1_01410	Hypothetical protein
Sp33	299510	MC1_01640	Putative toxin of toxin-antitoxin (TA) system
Sp34	888003	MC1_05085 **	Actin polymerization protein RickA
Sp35	589425	MC1_03370	Thiol:disulfide interchange protein dsbA
Sp36	230327	MC1_01215	Prolyl endopeptidase
Sp37	637085	MC1_03670	Hypothetical protein
Sp38	292360–292361	MC1_01595	S-adenosylmethionine:tRNA ribosyltransferase-isomerase
Sp39	912985	MC1_05235	Hypothetical protein (RARP-2)
Sp40	1279632	Intergenic	n/a
Sp41	995818	Intergenic	n/a
Sp42	372020	MC1_02055 **	GTP-binding protein LepA
Sp43	651603–651604	MC1_03735	ADP, ATP carrier protein
Sp44	868641	MC1_04970	HAD-superfamily hydrolase
Sp45	761156	MC1_04295	Microcin C7 resistance protein
Sp46	852817	MC1_04870	Methylated-DNA-protein-cysteine methyltransferase
Sp47	856486	MC1_04920	Hypothetical protein
Sp48	243782–243780	MC1_01300	DNA repair protein RecN
Sp49	687339	MC1_03930	Hypothetical protein
Sp50	1158028	MC1_06730	Hypothetical protein
Sp51	888088	MC1_05085 **	Actin polymerization protein RickA
Sp52	346470	Intergenic	n/a
Sp53	793762	MC1_04525	Hypothetical protein
Sp54	1258878	MC1_07285	Hypothetical protein
Sp55	1245896	MC1_07200	tig Trigger factor
Sp56	1243741–1243742	Intergenic	n/a
Sp57	1004249–1004250	Intergenic	n/a
Sp58	1273429	MC1_07360	NAD(P) transhydrogenase subunit alpha
Sp59	1181227	Intergenic	n/a
Sp60	1158028	MC1_01115	Hypothetical protein
Sp62	350030–350031	MC1_01915	Cytochrome c oxidase assembly protein
Sp63	109070	MC1_00650 **	Surface cell antigen (Sca2)
Sp64	314932	MC1_01745 **	Ankyrin repeat-containing protein (RARP-1)
Sp65	726967	Intergenic	n/a
Sp66	615509–615510	MC1_03545	Hypothetical protein
Sp71	371351	MC1_02055 **	GTP-binding protein LepA
Sp72	83786	MC1_00525	Stage 0 sporulation protein J
Sp73	655844	MC1_03745	Putative transcriptional regulator
Sp74	991759–991760	MC1_05745	Hypothetical protein
Sp75	251100	MC1_01335	Ankyrin repeat-containing protein
Sp76	9674	Intergenic	n/a
Sp78	672659	Intergenic	n/a
Sp79	65481	Intergenic	n/a
Sp80	1210788–1210789	MC1_07040	Outer membrane protein OmpA
Sp81	365135	MC1_02000	Cytochrome b
Sp82	549578–549579	MC1_03115	Cytochrome c oxidase polypeptide
Sp83	480829	MC1_02715	Hypothetical protein
Sp84	689140	Intergenic	n/a
Sp85	514488	Intergenic	n/a
Sp88	241435	MC1_01295	Thermostable carboxypeptidase
Sp90	1127301	MC1_06610	Hypothetical protein
Sp91	82796	MC1_00515	16S rRNA methyltransferase GidB
Sp92	1229489–1229490	MC1_07110	17 kDa surface antigen
Sp93	1223170	MC1_07070	Undecaprenyl-phosphate alpha-N-acetylglucosaminyltransferase
Sp94	774831	Intergenic	n/a
Sp95	902617	MC1_05150	Patatin b1
Sp96	561640–561641	MC1_03180	Hypothetical protein
Sp97	641129–641130	MC1_03685	miaA tRNA delta(2)-isopentenylpyrophosphate transferase
Sp98	34100–34101	MC1_00220	Putative methyltransferase
Sp99	1104365	Intergenic	n/a
Sp100	375061	Intergenic	n/a
Sp101	152889–152890	Intergenic	n/a
Sp102	406474–406475	MC1_02260	DNA mismatch repair protein MutS
Sp103	662735	MC1_03780	Hypothetical protein
Sp104	1161553	MC1_06745	Hypothetical protein
Sp105	593543–593544	MC1_03405	Acylamino acid-releasing protein
Sp106	1045462–1045463	MC1_06065	Outer membrane protein OmpB
Sp107	531709	MC1_03025 **	ampG protein
Sp108	1177263	MC1_06810	F0F1 ATP synthase subunit beta
Sp109	54288–54289	MC1_00370 **	Chaperone ClpB
Sp111	854916	Intergenic	n/a
Sp112	55657	MC1_00370 **	Chaperone ClpB
Sp113	319455	MC1_01760	Histidine kinase sensor protein
Sp114	765596	MC1_04335	Ribonuclease D
Sp115	229548	Intergenic	n/a
Sp116	314408	MC1_01745 **	Ankyrin repeat-containing protein (RARP-1)
Sp117	733347	MC1_04135	Hypothetical protein
Sp118	695571	Intergenic	n/a
Sp119	231259	MC1_01235	Prolyl endopeptidase
Sp120	27839	MC1_00155	Hypothetical protein
Bp2	756531	MC1_04275	Hypothetical protein

Spontaneous rifampicin resistant mutants: Sp4-5, 69–70, 86, Bp1. Clones that did not expand: Sp16, 32, 61, 67–68, 77, 87, 89, 110. Mapping for Sp12 revealed two different insertion sites and was not included in the list above.

** Indicates more than one isolated transposon mutant/gene. n/a, not applicable.

## Discussion

A critical barrier to identifying and characterizing virulence factors in obligate intracellular bacterial pathogens has been the inability to easily manipulate their genomes. In this study, we sought to overcome this barrier and harness recent advances in rickettsial genetics to build a library of transposon mutants of the SFG *Rickettsia* species, *R*. *parkeri*. We streamlined previous protocols to introduce a *mariner*-based transposon into the *R*. *parkeri* genome and isolated 106 transposon insertion mutations. Our study represents the first such transposon mutant library in this species, and the most extensive reported library in the rickettsiae.

In our study, we selected for mutants that showed an altered plaque size phenotype in infected host cell monolayers. Transposon mutations may cause a small plaque phenotype due to any number of defects, including: poor bacterial replication, reduced access to or survival within the cytosol, impaired cytosolic actin-based motility, and defective cell-to-cell spread. It was thus not surprising that we identified genes with a diverse set of predicted functions. Many genes with products predicted to perform bacterial-intrinsic functions (e.g. DNA replication) were identified and are expected to indirectly influence host-pathogen interactions through their role in bacterial growth and division. Other genes had more direct connections to the infectious life cycle and were further characterized in our recent studies to reveal their specific functions in intracellular infection [14,20]. For example, we previously described transposon mutations that disrupt the *rickA* (Sp34) and *sca2* (Sp2) genes and showed that these gene products are required for two independent phases of *R*. *parkeri* actin-based motility [20]. We also identified a transposon insertion (Sp19) in *sca4* gene and showed this encodes a secreted effector that promotes cell-to-cell spread [14].

Other genes mutated in this screen have been suggested to play critical roles during the infectious life cycle of other *Rickettsia* species but have yet to be characterized in *R*. *parkeri*. For example, we isolated transposon insertion mutants in the *ompA* (Sp80) and *ompB* (Sp106) genes, encoding the outer membrane proteins OmpA and OmpB. Work with SFG species *R*. *conorii* and *R*. *rickettsii* showed that OmpA and OmpB may regulate adhesion to and/or invasion of host cells [22–25]. However, some of this work relied on expression of these proteins in other bacterial species because mutants were not available. Interestingly, targeted knockout of *ompA* in *R*. *rickettsii* via an LtrA group II intron retrohoming system revealed no clear requirement for OmpA in invasion [26], suggesting it alone is not necessary. This highlights the importance of studying loss-of-function mutants to reveal gene function. The fact that *ompA* and *ompB* mutants exhibit a small plaque phenotype suggest additional functions of these proteins, putative indirect effects of the truncated products, or simply a need for efficient invasion of neighboring cells after host cells lyse during plaque development. Future work on these mutants should help reveal the relative importance of these proteins during invasion and/or other stages of the *R*. *parkeri* life cycle.

Our screen also revealed genes for which no specific role has been ascribed during the infectious life cycle, although the sequence of their protein products suggests a role in interaction with host cells. These proteins include some with eukaryotic-like motifs such as ankyrin repeats, which often mediate protein-protein interactions [27], and are a common motif in secreted bacterial effector proteins or virulence factors that target host pathways [27,28]. In particular, mutations in genes encoding *R*. *parkeri* orthologs of RARP-1 and RARP-2 from *R*. *typhi* (accession numbers MC1_01745 and MC1_05235, respectively) were identified in our screen (Sp64, Sp116, and Sp39). Work in *R*. *typhi* has revealed that RARP-1 and RARP-2 are secreted into the host cell, but their precise functions remain unknown [29,30].

Another mystery in rickettsial biology relates to the functional importance of the putative type IV secretion system (T4SS) encoded in their genomes [31], which in other species is involved in translocating DNA, nucleoproteins, and effector proteins into host cells [32]. Strikingly, the *Rickettsia* T4SS has an unusual expansion of the VirB6-like genes (i.e. Rickettsiales vir homolog, *rvhB6*), which are predicted to encode inner membrane protein components at the base of the T4SS [30,31,33]. Interestingly, we isolated a strain with a transposon insertion mutation in the fifth VirB6-like gene, *rvhB6e* (Sp9). This mutant will prove useful to explore the function of the T4SS in rickettsial infection.

Finally, we identified 20 strains, each carrying a transposon insertion disrupting a gene encoding a hypothetical protein. One of these caused a big plaque phenotype, suggesting enhanced growth or cell-to-cell spread. Further study of these mutants has the potential to reveal the function of these uncharacterized gene products during rickettsial infection. We also identified 25 small plaque mutants with insertions in intergenic regions. In these cases, the mutant phenotype could be caused by transposon insertion into a promoter region that alters gene expression. These mutants may represent tools for exploring gene regulation during the *R*. *parkeri* life cycle.

Overall, our mutant collection provides an important resource that can be used to uncover key bacterial players that regulate rickettsial interactions with their host cells. This will also allow for more direct analysis of gene function in the rickettsiae without the reliance on introducing genes into heterologous organisms. This forward genetics approach has the potential to reveal new insights into rickettsial biology and pathogenesis; however, limitations remain. For example, because the rickettsiae are obligate intracellular pathogens, screens such as these are unlikely to reveal genes that are essential for invasion or intracellular growth. Therefore, we cannot necessarily assess the relative importance of genes not identified in forward genetic screens. Additionally, the reported protocol still has limits with regard to electroporation efficiency and recovery on host cells, which makes it harder to adapt for large-scale mutagenesis work. Continued optimization of the protocols we report—by further characterizing and enhancing transformation efficiency and bacterial viability—will help investigators expand the available toolkit to generate more *Rickettsia* mutants. Additional advancements in rickettsial genetic methods will also be necessary to complement our work and more effectively probe the molecular mechanisms of all genes whose products may control critical host-pathogen interactions.
